# Bioinspiring *Chondrosia reniformis* (Nardo, 1847) Collagen-Based Hydrogel: A New Extraction Method to Obtain a Sticky and Self-Healing Collagenous Material

**DOI:** 10.3390/md15120380

**Published:** 2017-12-04

**Authors:** Dario Fassini, Ana Rita C. Duarte, Rui L. Reis, Tiago H. Silva

**Affiliations:** 1 3B’s Research Group—Biomaterials, Biodegradables and Biomimetics, University of Minho, Headquarters of the European Institute of Excellence on Tissue Engineering and Regenerative Medicine, AvePark, Barco, Guimarães 4805-017, Portugal; dario.fassini@gmail.com (D.F.); aduarte@dep.uminho.pt (A.R.C.D.); rgreis@dep.uminho.pt (R.L.R.); 2 ICVS/3B’s PT Government Associated Laboratory, Braga/Guimarães 4710-057, Portugal

**Keywords:** marine collagen, hydrogel, collagen rheology, marine sponge GAG, marine biomaterials, *Chondrosia reniformis*

## Abstract

Collagen is a natural and abundant polymer that serves multiple functions in both invertebrates and vertebrates. As collagen is the natural scaffolding for cells, collagen-based hydrogels are regarded as ideal materials for tissue engineering applications since they can mimic the natural cellular microenvironment. *Chondrosia reniformis* is a marine demosponge particularly rich in collagen, characterized by the presence of labile interfibrillar crosslinks similarly to those described in the mutable collagenous tissues (MCTs) of echinoderms. As a result single fibrils can be isolated using calcium-chelating and disulphide-reducing chemicals. In the present work we firstly describe a new extraction method that directly produces a highly hydrated hydrogel with interesting self-healing properties. The materials obtained were then biochemically and rheologically characterized. Our investigation has shown that the developed extraction procedure is able to extract collagen as well as other proteins and Glycosaminoglycans (GAG)-like molecules that give the collagenous hydrogel interesting and new rheological properties when compared to other described collagenous materials. The present work motivates further in-depth investigations towards the development of a new class of injectable collagenous hydrogels with tailored specifications.

## 1. Introduction

Collagen is the most abundant protein of the extra cellular matrix and can be found in all Phyla [1] with the remarkable exception of Placozoa [2] and Rotifera [3]. Collagen is the natural cell scaffolding and has several domains that bind proteoglycans [4,5], growth factors [6] and other cell signalling molecules [7,8]. In this view, it is regarded as an ideal material for many applications dealing with human health and wellbeing, including regenerative medicine [9,10,11,12,13,14,15,16]. Although collagens sources are still mainly derived by mammals (bovine and porcine), scientists and entrepreneurs are being challenged by specific concerns regarding zoonosis, potential immunogenic reactions [14,15] as well as ethical and religious concerns [17]. For those reasons marine organisms in the last years have gained much popularity as potential alternative source of safer and more acceptable source of collagen [17].

The potential applications of collagens extracted from marine organisms include Tissue Engineering and Regenerative Medicine (TERM); wound healing, drugs and gene delivery/carrier; cosmetic, food industry as well as nutraceutics [17]. Once sufficiently purified, collagen used in Tissue Engineering and Regenerative Medicine is generally processed in order to obtain solid scaffolds or highly hydrated scaffolds; also knew as hydrogels. One of the most common steps during collagen processing consists in the introduction of artificial crosslinks in order to stabilize the structure and to control its degradation rate once grafted. Crosslinks can be achieved by: physical [18,19]; chemical [20,21,22,23,24,25,26,27]; enzymatic [28] treatments or, less frequently, by combining two different approaches [29,30].

*Chondrosia reniformis* Nardo, 1847 is a common marine demosponge that lives in the shallow costs of the Mediterranean Sea and the South-West cost of the Atlantic Ocean [31]. The skeleton elements of the class Demospongiae are composed of an inorganic and an organic component. The inorganic component consists of a broad array of siliceous spicules [32] while spongin, collagen and chitin are the three constituents of the organic skeleton [33,34]. In contrast to most demosponges skeletons, which are constituted of spicules in association with one or more organic components, *C. reniformis* lacks both endogenous spicules and spongin/chitin elements. By contrast *C. reniformis* is particularly rich in collagen. This species has attracted the attention of scientists for the capability to modulate its mechanical properties by acting on the collagen crosslinks [35,36,37] and, since its high content, as an alternative source of collagen [11,38,39,40,41]. Intact fibrils, which resemble type I collagen [38], can be isolated using 4% EDTA [42], the alkali method described by Swaschtek and colleagues [11] or using a solution containing both EDTA and 2-mercaptoethanol based on a slightly modified protocol [37] firstly developed by Matsumura and coworkers for echinoderms [43]. *C. reniformis* collagen is particularly insoluble in acidic organic and inorganic media [38], and so far collagen/gelatin have been obtained only after trypsin digestion of sponge material [42]. Nonetheless, a recently reported study refers the use of water acidified with CO_2_ for the successful extraction of collagen/gelatin from this sponge species [40,41], in a mixture with other unidentified compounds.

The aim of the present work was to establish a new method to increase the amount of collagen that can be extracted from the marine sponge *C. reniformis* preserving intact fibrils and able to directly produce a collagenous hydrogel. The extraction was performed separately for the two different sponge regions: the ectosome (Ec; the outer cortical layer), and the choanosome (Ch) that constitutes the main bulk of sponge. Independent collagen extractions from the two regions were run in parallel in order to evaluate potential differences in the characteristics of hydrogels given that type IV collagen is more abundant in the ectosome than in the choanosome [44].

## 2. Results

### 2.1. Collagen Extraction

**Scheme 1:**
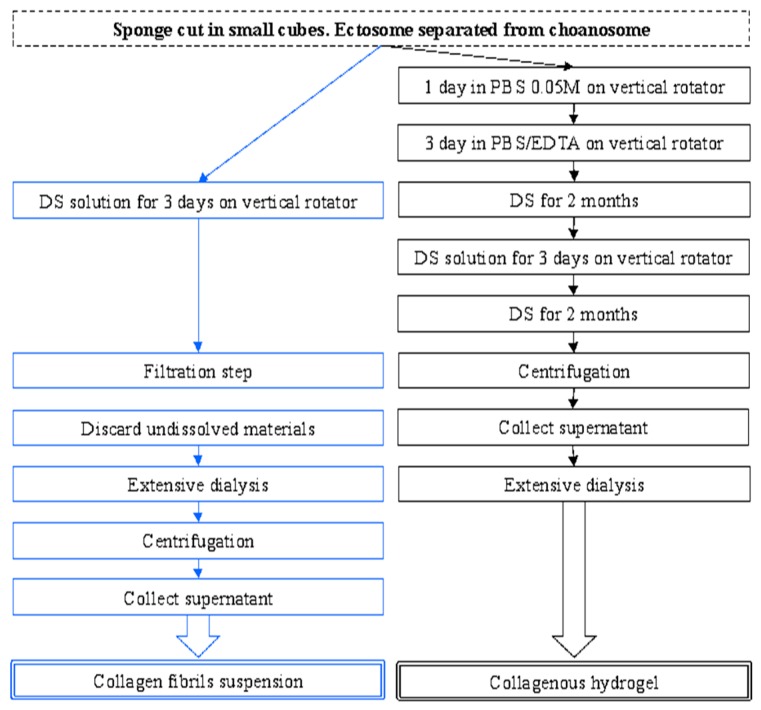
Schematic representation of the extraction procedure as described in Fassini et al. 2014 [37] (blue squared text, left side) and the new extraction procedure (black squared text, right side). The initial step is the same for both the treatments (broken line); different treatments with similar aims are put on the same line in order to highlight the similarities and differences between the two extraction procedures. The final results of the two protocols is showed in the double bounded squares. PBS = phosphate buffer saline; EDTA = ethylenediaminetetraacetic acid; DS = disaggregating solution.

In comparison with the method described by Fassini et al. [37] and herein referred as the reference procedure, a pre-treatment step in phosphate buffer saline/ethylenediaminetetraacetic acid (PBS/EDTA) followed by a significantly longer period of incubation in disaggregating solution (DS) was necessary to directly obtain the collagenous hydrogels (Scheme 1).

**Figure 1:**
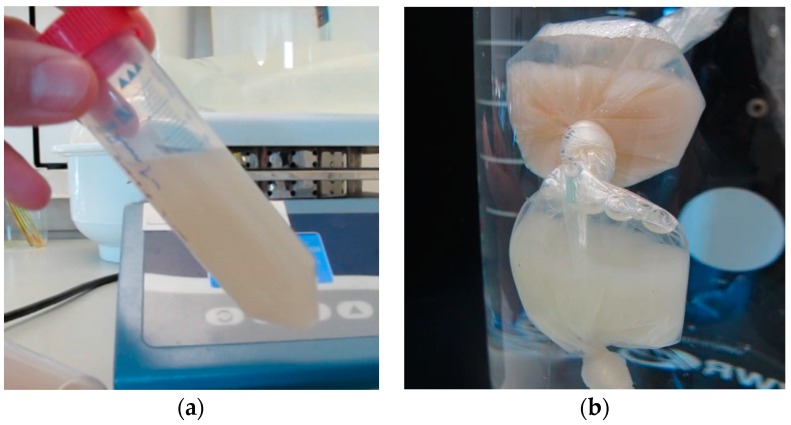
Image of a choanosome sample before (**a**) and during (**b**) the dialysis step.

During the collagen extraction process, sponge pieces significantly shrink and take on a more whitish color during the treatment with EDTA. While still in DS the material has a homogeneous liquid consistency similar to that observed during the reference procedure (Figure 1a), during the dialysis the new material precipitates (Figure 1b).

At the end of the dialysis process, the collagenous materials obtained either from Ch or Ec consist of a sticky jelly-like body (see Video S1 in Supplementary Materials). The obtained materials were dramatically different from that where the pre-EDTA treatment was skipped. While the latter material was still liquid, the former material was almost solid and able to adhere moderately to plastic and nitrile materials (such as containers and gloves) and remarkably also onto wet surfaces. The materials showed also evident self-healing properties being able to regenerate its integrity after it has been disrupted. Following repeated manipulations aimed to stretch the collagenous gel-like body, it recovers the initial shape when the force is withdrawn (see Video S1 in Supplementary Materials). Furthermore, the material extracted could be concentrated by centrifugation or diluted by adding a water-based buffer and generous shaking.

A large variability in terms of concentrations (dry weight/mL) was observed. The range was comprised between 16.8 and 6.92 mg/mL. Higher concentrations were obtained from Ec samples given the fact that Ec is characterized by a higher abundance of both non fibrillar [44] and fibrillar [45,46] collagen than choanosome. In this view the higher yield of extracted material from Ec (126–109 mg/g of fresh sponge tissue) compared to Ch (77–50 mg/g of fresh sponge tissue) was not surprising. Remarkable differences in the concentrations were also observed among different hydrogels obtained from the same batch (Table 1, confront batch 2a and 3a). The variability is likely produced during the dialysis step. Indeed the use of dialysis membrane tubes does not allow to control the water uptake. Significant discrepancies were discovered between the total dry mass and the collagen/protein content (Table 1).

Collagen content of Ch batches extracted from different specimens was not directly related to the dry content and varied between 7 and 17 µg/mL while proteins range was comprised between 66 and 133 µg/mL (Table 1), being the latter quantified as smaller than 2% of the extract and collagen as roughly 10% of the total protein mass. This suggests that some other compounds like Glycosaminoglycans (GAG, see below) and, possibly, salts were present in the extracts. Moreover, it is highly plausible that sponge collagen did not react extensively with the chemicals in either the both Sircol and BCA assay due to the presence of non-solubilized collagen fibrils, the high HLys content and the presence of attached glycans [39].

### 2.2. Sodium Dodecyl Sulphate Polyacrylamide Gel Electrophoresis (SDS-PAGE)

**Figure 2:**
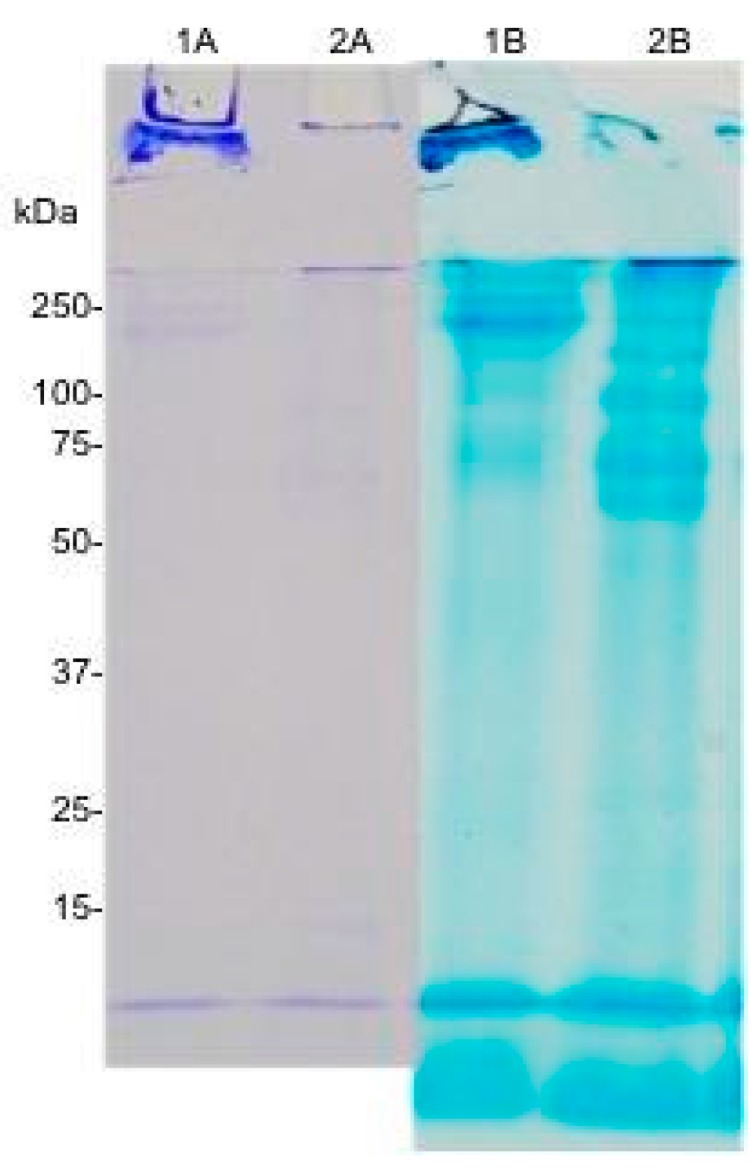
SDS-PAGE of ectosome (Ec) extract after one month (lane 1A, 1B) or two months (lane 2A, 2B) in DS stained with Coomassie R-250 (1A, 2A) or alcian blue (1B, 2B). A volume corresponding to 250 µg of material was added to each well.

The obtained extracts were submitted to electrophoretic analyses to better characterize their biochemical composition. Conventional collagen extracts and most of proteins are easily stained with Coomassie dye also at rather low concentrations. However, this was not the case of *C. reniformis* collagens, which were detected only after staining the gels with alcian blue (Figure 2).

Indeed, at 1 mg/mL, a concentration generally sufficient to well resolve the characteristic chain composition of fibrillar collagens, sponge collagen bands were rather evanescent when stained with R-250 Coomassie, but well visible when stained with alcian blue.

Comparing the protein profile one month after the start of the incubation (Figure 2—compare lanes 1A, 1B) and at the end of the second month (Figure 2—compare lanes 2A, 2B) in the DS, it is possible to see the effects of a prolonged incubation. Most of the collagen after one month in the DS is still in the form of large fibrils that cannot be solubilized and thus remain trapped in the stacking gel. However, after an additional month in DS, collagen fibrils are almost completely solubilized in their single components. In our gels we observed at least seven bands in the high molecular weight region plus a broad band that runs faster than the frontline. While some bands did not change over time (i.e., at one and two months of incubation), others appeared only after the complete incubation in DS solution, suggesting a significant increase in the solubility of collagen fibrils. Indeed, the fibrils trapped in the stacking gel (Figure 2—lane 1B), which are likely associated in small bundles as a result of interactions with other proteins, completely disappear between month 1 and 2 (Figure 2—lane 2B). On the other hand, we found a new band that was blocked at the beginning of the stacking gel. Considering its very high molecular weight, together with results detailed in Section 2.3 (below), i.e., the sensitivity to both Coomassie and alcian blue dyes as well as its resistance to a long pepsin digestion, this band was likely to have originated from the presence of isolated collagen fibrils.

### 2.3. Effect of Pepsin Digestion

Samples were treated with pepsin in order to evaluate whether some proteins or the collagen telopeptides were responsible for the gel consistency of the obtained materials and to confirm the collagenous materials of the bands observed in the SDS-PAGE. 

**Figure 3:**
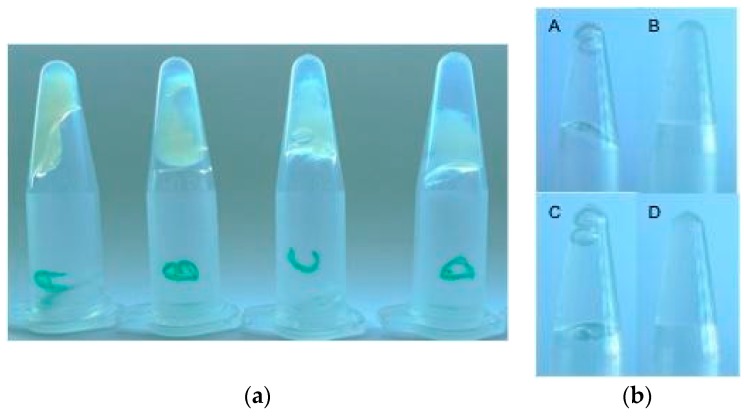
Effects of pepsin digestion on choanosome (Ch) collagen extract. (**a**) Images of the materials soon after the digestion/control treatment; and (**b**) meniscus details of the supernatants obtained after the removal of the insoluble component. Tubes legend: A = Ec digested; B = Ec control; C = Ch digested; D = Ch control.

We found that there were no qualitative macroscopic differences between untreated extracts and the ones treated with pepsin; on the other hand, the supernatant obtained after a rapid centrifugation, was significantly different. Supernatants of undigested samples formed a nearly 90° meniscus while the digestion caused a change in the meniscus angle that was >90° (Figure 3).

**Figure 4:**
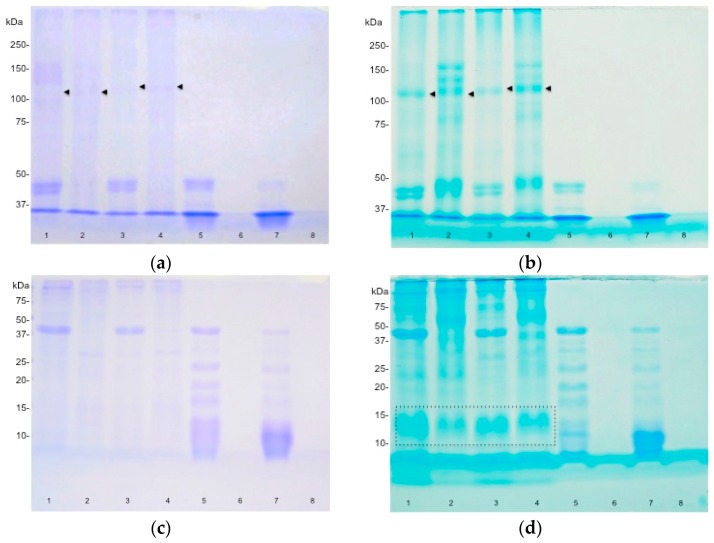
7.5% (**a**,**b**) and 15% (**c**,**d**) sodium dodecyl sulphate-polyacrylamide gel electrophoresis (SDS-PAGE) stained with R-250 Coomassie (**a**,**c**) and alcian blue (**b**,**d**) of digested and undigested samples. Lanes 1/5 = digested Ec; 2/6 = undigested Ec; 3/7 = digested Ch; 4/8 = undigested Ch. Lanes 1–4 = pellet; 5–8 = supernatant. Pellets obtained after centrifugation were resuspended in 800 µL of loading buffer and freeze-dried supernatants in 80 µL; 40 µL of the resulting dispersions were added in each well. Arrowhead = presumptive collagen; broken line rectangle = possible glycan.

SDS-PAGE analysis was carried out on all the samples, including the supernatants obtained after the removal of the insoluble parts. In terms of composition, the material obtained from Ch was less complex than that extracted from Ec (Figure 4, compared undigested Ec and Ch).

Moreover, the SDS-PAGE profile of collagens treated with pepsin revealed a significant difference with the untreated ones. In particular, several bands in the higher (>100 kDa) molecular weight appeared in the Ec (compare lane 12 s—tained with Coomassie of Figure 4) suggesting that pepsin treatment was able to solubilize different proteins. Both Ec and Ch-solubilized collagens had a lower molecular weight when treated with pepsin suggesting that the enzyme was able to remove collagens telopeptides. Our data suggests that a slight difference between Ec and Ch collagens does exist, while both seems to be homotrimeric. Pepsin treatment seems to increase the solubility and the Coomassie sensitivity of other kind of collagens present in the ectosome. Considering the evident effect of pepsin treatment on the supernatant (Figure 4) it is likely to be that some pepsin-sensitive molecules not stained with the employed dyes were present in the samples.

The combined analysis of Coomassie/alcian blue staining (Figure 4) revealed the presence of a glycan that, since it was not found in the supernatant, should be stably associated with the material itself.

**Figure 5:**
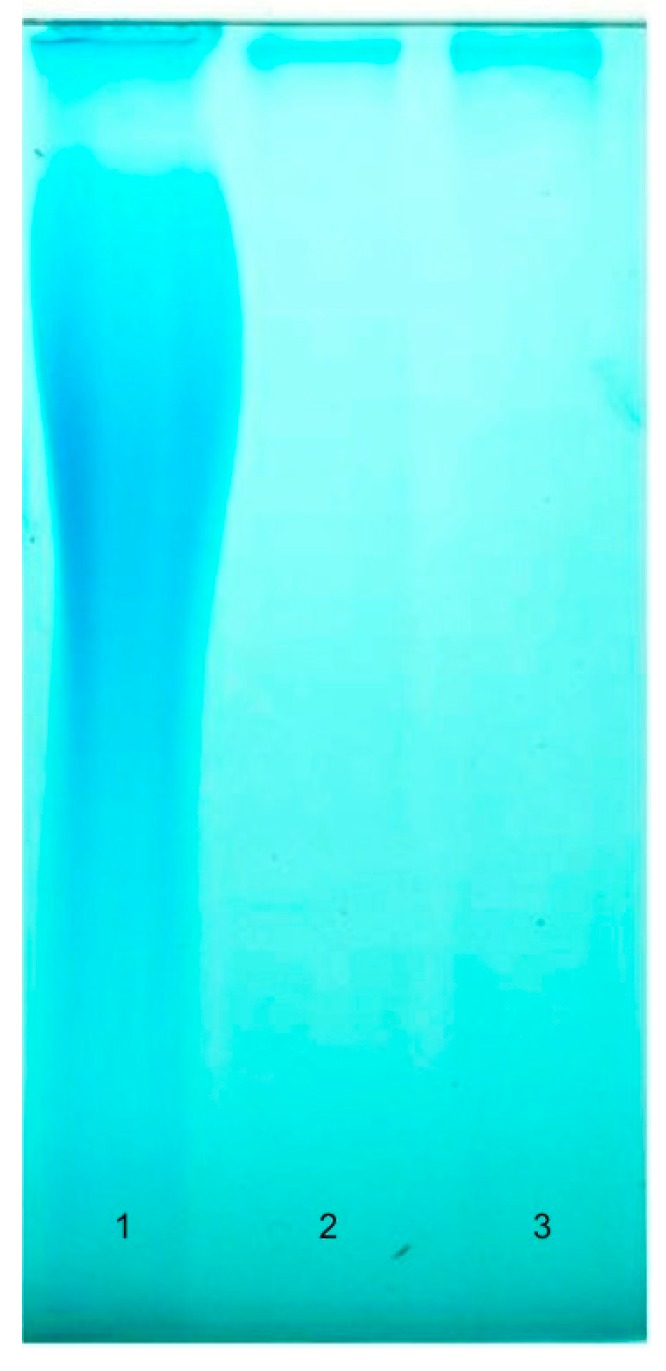
10% Tris/borate/EDTA polyacrylamide gel electrophoresis (TBE-PAGE) of glycosaminoglycans (GAG) extracted from choanosome (Ch; lane 2) and ectosome (Ec; lane 3), after staining with alcian blue. A similar quantity (170 µg) of shark chondroitin sulfate was loaded into lane 1 as reference.

The materials obtained from either Ch or Ec were completely dissolved after few hours when treated with papain at 50 °C. After the precipitation of GAGs and the following freeze-drying step, quite a large amount of material was obtained. About 3 mg per mL of starting materials (Ec 14.4 mg/mL and Ch 10.6 mg/mL) were recovered; however, when run trough Tris/borate/EDTA polyacrylamide gel electrophoresis (TBE-PAGE), only a small band was detected (Figure 5).

Neither of the bands were susceptible to an extensive incubation with Chondroitinase ABC (data not shown), and thus the nature of this molecules could not be assessed.

### 2.4. Fourier-Transformed Infrared Spectroscopy (FTIR)

**Figure 6:**
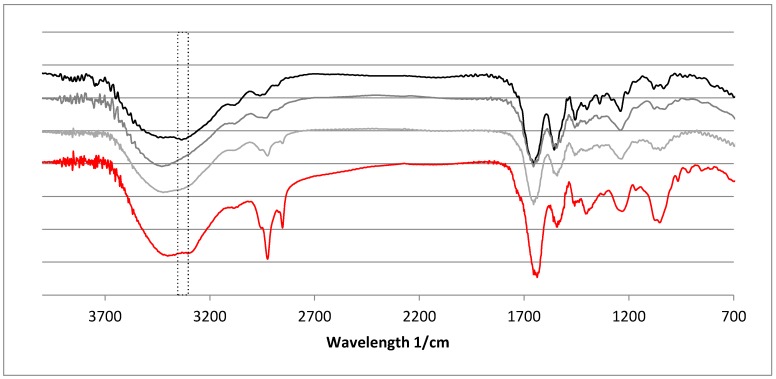
FTIR spectra of Sigma bovine type I collagen (black); Ec (dark grey); Ch (light grey); Ch supernatant (red). Broken line rectangle indicate Amide A region. Absorbance values have been shifted to facilitate the reading.

Collagens extracted from both the Ec and Ch were characterized in their IR absorption spectra. Overall, the obtained spectra (Figure 6) are in accordance with the results described in literature [38,42].

In more detail, the analysis of the obtained FTIR peaks indicated the presence of significant differences in the amide A region for both Ec and Ch. The asymmetric NH_2_ stretching peak in the Ec samples was difficult to identify due to a nearby strong peak associated with the presence of hydrogen bonds. This result is again in accordance to the FTIR collagen spectra describe by Garrone and coworkers [42]. All other major peaks, listed in Table 2, correspond quite well type I collagen (Sigma Aldrich, Merck KGaA, Darmstadt, Germany), used as reference material. 

The comparison of the FTIR spectra with Type I collagen (Sigma Aldrich, Merck KGaA, Darmstadt, Germany) revealed a substantial similitude in the peaks; finally the ratio of absorbance measured at 1235 cm^−1^ and 1450 cm^−1^, which were all >0.9, suggests the presence of native collagen triple helix in all the samples [49].

### 2.5. Rheology

**Figure 7:**
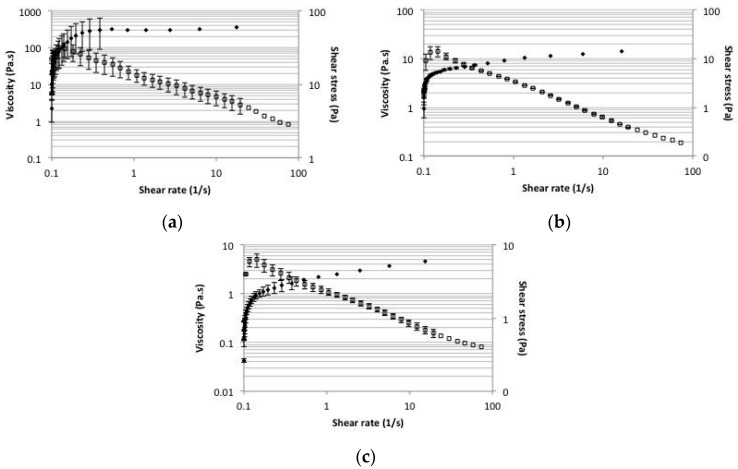
Flow curves of ectosome (Ec) and choanosome (Ch) subjected to increasing shear rates. (**a**) = Ec 14.4 mg/mL; (**b**) = Ch 9.6 mg/mL; (**c**) = Ch 8.2 mg/mL (Ch 9.6 mg/mL after centrifugation and resuspension). Square = viscosity; diamonds = shear stress. Values are expressed as the average of three repeated experiments; bars = standard deviation.

Considering the unusual properties of the obtained collagenous materials and to better understand their mechanical performance the rheological properties of the collagenous materials isolated from both Ec and Ch were characterized using a rheometer. Both materials display a clear shear thinning behavior (Figure 7a,b respectively).

In all the samples the viscosity, which at low shear rates tends initially to increase, decreased almost constantly at higher shear rates. The sample extracted from the Ec was much more concentrated than the one obtained from the choanosome, which partially justifies the differences observed in absolutes values when viscosity is considered. Additionally, we cannot exclude that differences in the composition may also be responsible for the observed disparities in the viscosity values.

Figure 7b,c presents the results of the same Ch sample before and after a centrifugation/resuspension step. In this step, the sample was diluted in dH_2_O 2:1, mixed thoroughly, centrifuged (6600 g for 1 min) to concentrate the collagenous part and then brought to the initial volume using dH_2_O. Upon this procedure it is worth remarking that a significant amount of soluble material was extracted. Indeed, the dry weight decreased from 9.6 mg/mL to 8.2 mg/mL. This treatment revealed that the soluble molecules present in the supernatant play an important role in the rheological properties as their absence brings a significant change in the response of the material (Figure 7). In particular, the treatment decreased the viscosity at low shear strain and the overall shear thinning behavior.

**Figure 8:**
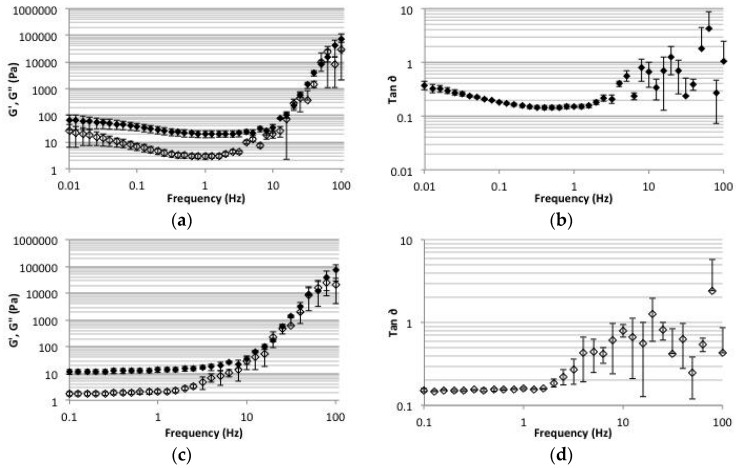
Storage and loss modulus measurements of choanosome (Ch) and ectosome (Ec) materials. Changes in the storage (G′, closed symbols), and loss (G″, open symbols) moduli at increasing frequency (**a**,**c**) and loss tangent (**b**,**d**) of Ec 14.4 mg/mL (**a**,**b**) and Ch 9.6 mg/mL (**c**,**d**). Values are expressed as the average of three repeated experiments; bars = standard deviation.

The gel nature of the collagenous materials was confirmed by analyzing the storage (G′) and the loss modulus (G″) at frequency between 0.1 and 100 Hz. The oscillatory measures provide relevant information on the molecular arrangement of the network of the gel and allowed the determination of the solid-like (elastic) and liquid-like (viscous) regions of the material. In the frequency range studied, the gel formed by the choanosome-derived material is in the plateau zone of the viscoelastic region, i.e., for frequencies lower than 1 Hz both G′ and G′’ are nearly constant (Figure 8).

Furthermore, in this region the storage modulus is higher than the loss modulus, which is a characteristic behavior of a viscoelastic fluid. The G″/G′ ratio, called the loss tangent (*Tan ∂*), gives a clear indication of the solid/liquid responses. Values lower than 1 indicate a more solid behavior while at values >1 a liquid-like behavior is prevalent. The curve shown in Figure 8 revealed that at 1.5 Hz the material starts to have a more liquid response. At higher values we observe clear crossing points at 20 and 80 Hz. This phenomenon is related with the entanglements of collagen chains [50]. The entanglements limit the mobility of the chains contributing to a stiffer structure, and hence a higher loss tangent. From our observations the *Tan ∂* values suggest that the material retained a more solid response also at high frequencies. The storage modulus of Ec was also higher than the loss modulus, at low frequencies (Figure 8).

Here we noticed an apparent decrease in both G′ and G″, with the latter slightly more pronounced than the former as demonstrated by the *Tan ∂* values between 0.01 and 0.15 Hz. Ch samples (similarly to the Ec ones) at higher frequencies tend to remain at *Tan* ∂ values lower than 1 although rapid changes in the *Tan ∂* values have been observed.

**Figure 9:**
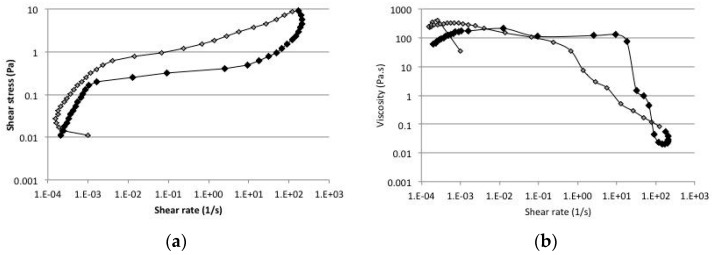
Ramp-up and ramp-down experiment showing the thixotropic property of the collagenous materials. Shear stress/rate (**a**) and viscosity/shear rate (**b**) curves of coanosome (Ch) 9.6 mg/mL in a ramp up/down experiment. Open grey symbols = ramp up; closed black symbols = ramp down. Lines connecting the points have been added, the points represent the average values of three different experiments. Standard deviations bars have been omitted to facilitate the reading of the graphs.

Following these observations, which suggest that the materials have a thixotropic behavior, specific rheological tests were further performed to confirm it. The thixotropic behavior of the Ch sample, less complex in terms of composition with respect to the Ec, was thus evaluated by the hysteresis loop obtained after a two-step experiment in which ramp-up and ramp-down experiments were performed. A large hysteresis area, as it can be observed in Figure 9, indicates the thixotropic properties of the gel, namely the ability of the gel to recover the initial structure after a certain stress is applied [51].

**Figure 10:**
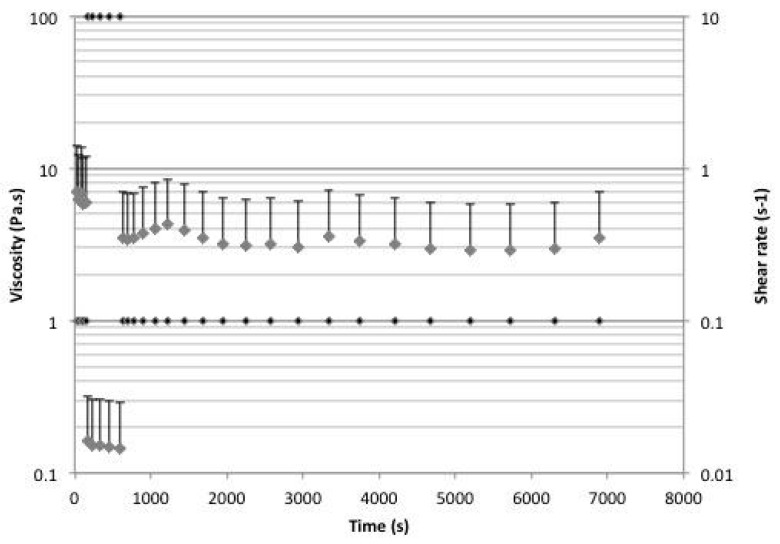
Viscosity values (grey dots) of coanosome (Ch) 9.6 mg/mL in a three-step experiment. Black dots = shear rate. Values are expressed as the average of three repeated experiments; bars = standard deviation.

The thixotropy of a material is also characterized by the ability to restore its original viscosity after a stress has been applied. Figure 10 presents the thixotropic behavior of the Ch sample in terms of viscosity in a three-step experiment.

We observed that during the ramp down phase, after a first rapid increase of viscosity, the values start to decrease and at very low shear rates are eventually much higher than the ones observed for higher shear rates, in which the viscosity should be around 10 Pa·s.

On the other hand, we found that after a steady increase of shear rate and a following decrease the material was not able to fully recovery its initial viscosity (Figure 10). However the evident initial loss of viscosity observed in the first phase of the experiments suggests that: small but prolonged shear stresses are able to modify the inner structure of the material suggesting that a much longer period to reach a viscosity equilibrium. In this view the viscosity values obtained after the stronger shear stress was removed are likely the real viscosity value of the material at the specific shear stress of 0.1 s^−1^.

## 3. Discussion

Collagen, which constitutes the bulk of the extracellular matrix of most of the animals, provides, among others features, the mechanical support of tissues and serves as well as anchoring points for cells and other molecules [52]. In this view, it is not surprising that it has long been proposed as a natural material for tissue engineering and regenerative medicine [53,54]. Among lower invertebrates, and sponges in particular, *C. reniformis* has been considered an interesting source of alternative and biocompatible collagen given its high collagen content [42] and for its interactions with silicic acids (Heinemann et al., 2007b). Moreover, among Porifera, *C. reniformis* is the reference model of dynamic collagenous tissues, which are characterized by the presence of labile and variable collagen crosslinks, and has been extensively investigated [35,36,37,55].

Standard acidic treatments, able to solubilize most of animal fibrillar collagens, have proven to be inefficient in the extraction of *C. reniformis* collagen and other dedicated protocols have been developed [11,37,41,42,56].

Our method is a substantial modification of the procedure described in Fassini et al. [34] and directly produced a collagenous hydrogel. In terms of FTIR spectra the collagenous hydrogels obtained were similar to data present in the literature. In particular, Ec collagen was very similar to the spectra provided by Garrone and coworkers [42], while Ch profiles were similar to what reported by Heinemann and coworkers [38]. Considering the fact that Garrone investigated the cortex (ectosome) while Heinemann investigated the whole sponge (where choanosome mass is predominant) our results are in line with both the previous publications. Our results provide evidence that some slow chemical modifications, able to increase the dissociation of collagen fibres, occur during the incubation in DS. Furthermore, we observed that the acidic pre-treatment with EDTA, which caused a significant bleaching and shrinkage of the mesohyl pieces, is necessary to obtain the sticky gel materials. Indeed the longest incubation period itself was not sufficient and the final product was a liquid collagen suspension identical to what described by Fassini et al. [37]. Given that acidic treatments do not influence the properties of *C. reniformis* collagen [38], the chelation activity on divalent ions of EDTA is most likely involved in the process. A solution of 4% EDTA is proven to influence the integrity of collagen bundles and to promote the formation of fibrils suspension [42], possibly by competing with the formation of the labile and calcium-dependent bonds that stabilize sponge collagen [36,37]. On the other side, our samples were exposed to significantly lower concentrations of EDTA, which apparently was not enough to dissociate the tissue. The significant increase in the solubilisation of the collagenous materials that was observed after two months is not clear. In the DS solution, EDTA and 2-mercaptoethanol are the active molecules that are mostly involved in the chelation of collagen stabilizing ions and in the reduction of disulphide bonds [43]. The fact that the solubilisation of the materials in reducing conditions occurred only after at least one month in DS, together with the relatively short half-life of 2-mercaptoethanol, signals that some other slow reaction occurred in the mixture during the incubation period. The nature of this possible reaction(s) is also still unclear. 

The materials obtained from the two different regions of the sponge have quite similar compositions although material obtained from Ch region has a higher collagen/non-collagen ratio than the one obtained from the Ec. The SDS-PAGE profile of digested samples revealed the presence of hydrolysis products, one single putative band for collagen at around 110 kDa and another three main bands of other unidentified proteins that appears to be stably associated with collagen. Indeed, proteins such as pepsin and peptides originating from the enzymatic digestion were more abundant in the supernatant obtained after the centrifugation than in the pellets while, on another hand, no proteins were found in the supernatant of untreated samples. Collagen extracted and solubilized from sponge was composed of a homotrimeric chain of around 105/115 kDa, the small molecular weight differences found between digested and undigested collagens suggest that the telopeptides are maintained during the extraction while differences between Ec and Ch might result from a different glycosylation level, different transcriptions, or by the presence of two different collagen types. Indeed, while the presence of type I collagen has been demonstrated in the sponge *Ircina fusca* [57], recent transcriptomic data from several sponges revealed the presence of the fibrillar type XI collagen [58].

The gel structure of both Ch and Ec was also transferred to the surrounding media and can be disrupted by the enzymatic digestion suggesting that some pepsin-sensitive protein is involved in the gel structure formation. However our efforts to investigate the nature of the molecules present in the supernatant were not conclusive.

The rheological properties of the materials obtained are difficult to compare with other described collagenous gels due to the complex nature of the extracts obtained. Moreover, *C. reniformis* collagen fibrils are rather different from type I collagen having an aspect ratio of 1:5000 [35], thus significantly inhibiting a proper comparison with the shorter and bigger mammal type I collagens.

Our data demonstrates the shear thinning and pseudoplastic properties of the materials, which exhibited gel like behavior. Moreover, considering the thixotropic behavior and the dramatic increase of its viscosity once the shear rate is decreased (i.e., recovering the viscosity properties when the external stimulus—stress—is removed), the possibility of modulating the mechanical properties while keeping the collagenous matrix unaltered suggests that the materials might be further investigated in order to design an injectable collagenous hydrogel.

In the recent years much effort has been focussed on collagenous hydrogels and on the strategies to control their biochemical and physical properties [9,59,60]. Hydrogels are particularly interesting for several tissue-engineering applications given their capability to provide a suitable substrate for cell growth and encapsulation and their high water content [61,62]. Other interesting and regarded properties of new designed hydrogels concerns their injectability and the capability to strongly adhere to wet surfaces [60], as well as the possibility to control their stiffness with simple changes in the pH, temperature or ionic strength, remaining inside the physiological boundaries of human tissues. In this sense, the extracts obtained with the methodology proposed in this work may see exciting developments in the future, namely in the context of advanced tissue regeneration therapies.

## 4. Materials and Methods

### 4.1. Sponge Sampling

*C. reniformis* specimens were collected at Paraggi (Eastern Ligurian Sea, 44°18′40′′ N, 9°12′46′′ E), transferred in a thermic bag to a tank filled with artificial sea water (Instant Ocean^®^, Blacksburg, VA, USA) and left rest for at least one night. Specimens were then frozen at −20 °C until further processing.

### 4.2. Collagen Extraction

Sponges were thawed and cut in small (1 × 1 × 1 mm) cubes with a scalpel; the outer brownish layer—the ectosome (Ec)—was separated from the inner yellowish core—the choanosome (Ch). Briefly 5 g of dry material (Ec or Ch) were transferred into two 50 mL tubes and in each tube 40 mL of 5× phosphate buffer saline (PBS, Sigma Aldrich, Merck KGaA, Darmstadt, Germany) were added before putting the samples on a vertical rotator at room temperature (RT). After 24 h, 10 mL of supernatant were removed and 100 mg ethylenediaminetetraacetic acid (EDTA, Sigma Aldrich) per gram of sponge material were added in the each tube. After three days on the vertical rotator, the pieces were transferred into a new tube containing the disaggregating solution (DS) composed of 0.1 M Tris, 0.5 M NaCl, 0.05 M EDTA, and 0.2 M 2-mercaptoethanol. Ten milliliters of DS/g of fresh sponge material were used, and samples were left in DS for two months at RT. The resulting mixtures were then centrifuged (15 min at 500 g) to remove undissolved parts and the extracts obtained were then extensively dialyzed against dH_2_O (ratio about 1:1000, two changes per day for 5 days) using a 14 kDa molecular weight cut off (MWCO) membrane tubing (Sigma Aldrich, Darmstadt, Germany) in order to remove all the 2-mercaptoethanol.

### 4.3. Collagen and Protein Quantification

Sircol Collagen Assay (Biocolor^®^, Carrick, UK) and BCA assay (MicroBCA protein detection kit, Pierce^®^, Thermo fisher Scientific, Waltam, MA, USA) were performed on the extracts from the choanosome according to the indications provided by the manufacturer, to quantify the amount of collagen and total proteins in the extracts, respectively. Readings were performed on a plate microreader (Synergy HT, Bio-Tek Instruments, Winooski, VT, USA), with calibration curve and concentrations of collagen/proteins being obtained by using the mean of at least three values for each standard/sample. Both Sircol and BCA assay were performed only on Ch hydrogels. The total amount of the extracted material was calculated by weighting standard amounts of freeze-dried material (mg/mL) and multiplying the values for the final volumes.

### 4.4. Enzymatic Digestions of Collagen and GAGs Extraction

In total 200 µL of extracts (in dH_2_O) obtained either from Ch or Ec were mixed with 200 µL of pepsin (porcine pepsin, Sigma) solution (2.1 mg/mL in 6 mM HCl). The digestion was performed for 72 h at 37 °C. After the digestion, the samples were observed and photographed capsize-down to check the effect of the digestion. Samples were then centrifuged for 15 min at 17,000 *g* to better separate the collagenous part from the above supernatant. Both supernatants and the pellets were freeze dried and stored at −80 °C for further experiments. Control samples were obtained in parallel in the absence of pepsin in order to exclude possible changes induced by the temperature, acidity and dilution.

*GAG extraction*: 3 mL of the extracted collagen was mixed with 5 mL of digestion solution (5 mg/mL of papain in 1 M NaBH_4_, 0.05 M NaOH) and placed at 45 °C overnight. Materials were completely dissolved after 2 h and GAG precipitation was performed slowly adding trichloroacetic acid (strong reaction) to reach a 50% vol/vol concentration according to the procedure reported by Nandini et al. [63]. The powders obtained were stored at RT for further characterization steps.

*GAGs digestion*: 10 mg of precipitated GAG were resuspended in 300 µL of PBS; 30 µL of 5 U/mL chondroitinase ABC (Sigma) were then added and enzymatic digestion was performed at 37 °C during 24 or 72 h. At each endpoint GAG were separated from the enzyme as described by Silva et al. [64]; briefly: 150 µL of the GAGs/enzyme mix were heated for 25 min at 70 °C and then centrifuged at 12,000 *g* for 25 min. The pellet was discarded while supernatant containing digested GAGs was recovered, freeze-dried and kept for further analysis.

### 4.5. Electrophoresis

#### 4.5.1. SDS-PAGE

All used reagents were purchased from Fluka (SDS-PAGE Kit, Thermo fisher Scientific, Waltam, MA, USA). Collagen batches were analysed after one month in DS through a fast dialysis (2 step at 1:10,000) and at the end of the second month in DS after the extensive dialysis above described. Samples were mixed 1:1 with loading buffer, boiled for 5 min at 100 °C and run in 1.5 mm 7.5% or 15% gels in a MiniProtean system (Biorad, Hercules, CA, USA).

Coomassie R-250 staining: gel was stained according to Laemmli [65]. Alcian blue staining: gels previously stained with Coomassie were treated with 12.5% thrichloroacetic acid for 30 min.; after 4 washes in dH_2_O gels were treated with 1% sodium metaperiodate in 3% acetic acid for 1 h. After numerous washes (until the discarded water was not reacting with 1% silver nitrate) gels were immersed in 0.5% alcian blue in 3% acetic acid for 4 h; excess of alcian blue was removed with extensive washes in 7% acetic acid. 

#### 4.5.2. Tris/Borate/EDTA Polyacrylamide Gel Electrophoresis (TBE-PAGE)

TBE-PAGE was prepared using 10× TBE buffer (Bioland) and reagents from Fluka (SDS-PAGE Kit). The 1.5 mm gels were run in a MiniProtean system (Biorad©), using 0.1 M Tris/borate, 1 mM NaEDTA, and pH 8.3 buffer. GAG (2 mg/mL) were mixed (1:5) with 2 M sucrose (Sigma) 0.02% bromophenol blue (Sigma) in 1× TBE. Gels were run at constant 60 V for 15 min and then at 150 V for 60 min at RT. After, gel was stained with 0.5% alcian blue in 2% acetic acid for 30 min; excess of staining dye was removed by rinsing the gel in 2% acetic acid for 30 min.

All the staining/washes steps were performed on an orbital shaker at room temperature.

### 4.6. FTIR

FTIR analysis was performed on freeze-dried samples. Dried materials were mixed with potassium bromide (Sigma), crushed with a pestle and processed into a thin pellet using a hand press. Spectra were recorded from 32 scans with a resolution of 2 cm^−1^ from 4000 to 700 cm^−1^ using a Shimadzu IR Prestige 21 Infrared spectrometer (Shimatzu, Kyoto, Japan).

### 4.7. Rheology

The rheological properties of the collagenous materials obtained after the dialysis process were investigated by means of a Kinexus Prot rheometer (Malvern instruments, Malvern, UK). Steady-state flow measurements were carried out using a controlled-stress rheometer fitted with parallel plate geometry with a 10-mm diameter (PU20 SR1740 SS) and 1-mm gap. The torque amplitude was imposed by using a logarithmic ramp of shear rate, in a range of 0.1 to 100 s^−1^. Rheological measurements in oscillatory frequency sweep strain controlled were performed in using the same parallel plate geometry with a 1 mm gap. Gel rheological behavior with storage/loss moduli and cross-over analysis was assessed at a constant 1% strain in a frequency region from 0.01 to 100 Hz.

All experiments were performed at a controlled temperature of 37 °C and results represent the average of three measurements.

## 5. Conclusions

We described a new method to extract collagen from the marine sponge *C. reniformis* resulting in sticky collagenous hydrogels. The materials obtained from two sponge regions, ectosome and choanosome, have singular rheological properties, with the observed shear thinning behavior seeming to depend on the presence of collagen fibrils and other associated proteins/GAGs. The weak interaction between those macromolecules have shown to be reversible and induce a fast recovery to the rest state of the viscosity after manipulation, suggesting their possible use as an injectable hydrogel medium for biomedical applications. The material obtained should be, however, further investigated to completely characterize its content and to fully address the role of the soluble compound(s). The data presented in this study represent a detailed starting point for further investigations aimed at producing tailored hydrogels with different interesting properties through a biomimetic approach.

## Figures and Tables

**Table 1:** Collagen and total proteins quantification of Ch batches determined by using Sircol and Bicinchoninic Acid (BCA) assays respectively. Batch 2a and 2b were obtained from an independent extraction starting from a different specimen.

Batch	Dry Weight (mg/mL)	Sircol (µg/mL)	BCA (µg/mL)
1	6.92	17.02	133.04
2	10.6	7.94	87.91
3	9.6	6.59	66.12

**Table 2:** List of representative FTIR peaks and the values obtained from FTIR analyses of Ch and Ec extracts. *S* = stretch; *B* = bend; *s* = symmetric; *as* = asymmetric; *HB* = hydrogen bonds.

Peak Wave Number/cm^−1^			Assignment	Reference
Sigma Type I	Ch	Ec		
3414	3414	3421	NH *S*, coupled with H-bond	[47]
3325	3331		*as* NH	[38]
3080	3082	3082	NH *S*	[48]
2951	2956	2962	CHx	[38]
2926	2924	2935	*as* CH_2_	[47]
	2852	2848	*s* CH_2_	[47]
1666	1654	1660	C=O	[38]
1651	1647	1654	C=O *S*/*HB* coupled with COO-	[47]
1556	1560	1556	NH *B* coupled with CN *S*	[38]
1545	1540	1544		
1450	1448	1450	Pyrrolidine ring	[49]
1458	1458	1458	CH_2_ *B*, CH_2_ *S*	[38]
1400	1396	1404		
1339	1340	1340	CN *S*	[38]
1235	1240	1240	CH_2_	[49]
1081	1082	1078	CO *S*	[38]
1033	1031	1033		

## Supplementary Materials

The following are available online at www.mdpi.com/1660-3397/15/12/380/s1, Video S1: demonstration of the adhesive and self-healing properties of the collagenous materials obtained from the choanosome.
